# Supplementary material for: CARD8 inflammasome activation during HIV-1 cell-to-cell transmission
Source: eLife. 2025 Jun 16;13:RP102676. doi: 10.7554/eLife.102676 (PMC12169848; doi:10.7554/eLife.102676)
Supplement: Figure 3—source data 1. [file elife-102676-fig3-data1.pdf]

Figure 3C: Cell-to-cell HIV infection induces CARD8-dependent inflammasome activation in monocyte-derived macrophages

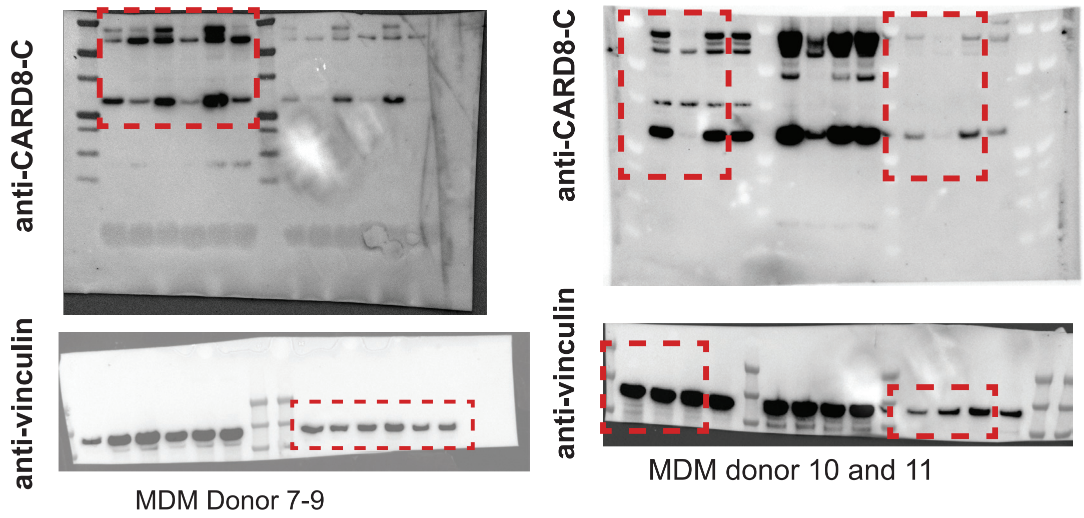

Uncropped Western Blots: Multichannel images shown are a merge of chemiluminescent and colorimetric images. Dashed red box indicates lanes used for figure
