# Supplementary figures and images for: CARD8 inflammasome activation during HIV-1 cell-to-cell transmission
Source: eLife. 2025 Jun 16;13:RP102676. doi: 10.7554/eLife.102676 (PMC12169848; doi:10.7554/eLife.102676)

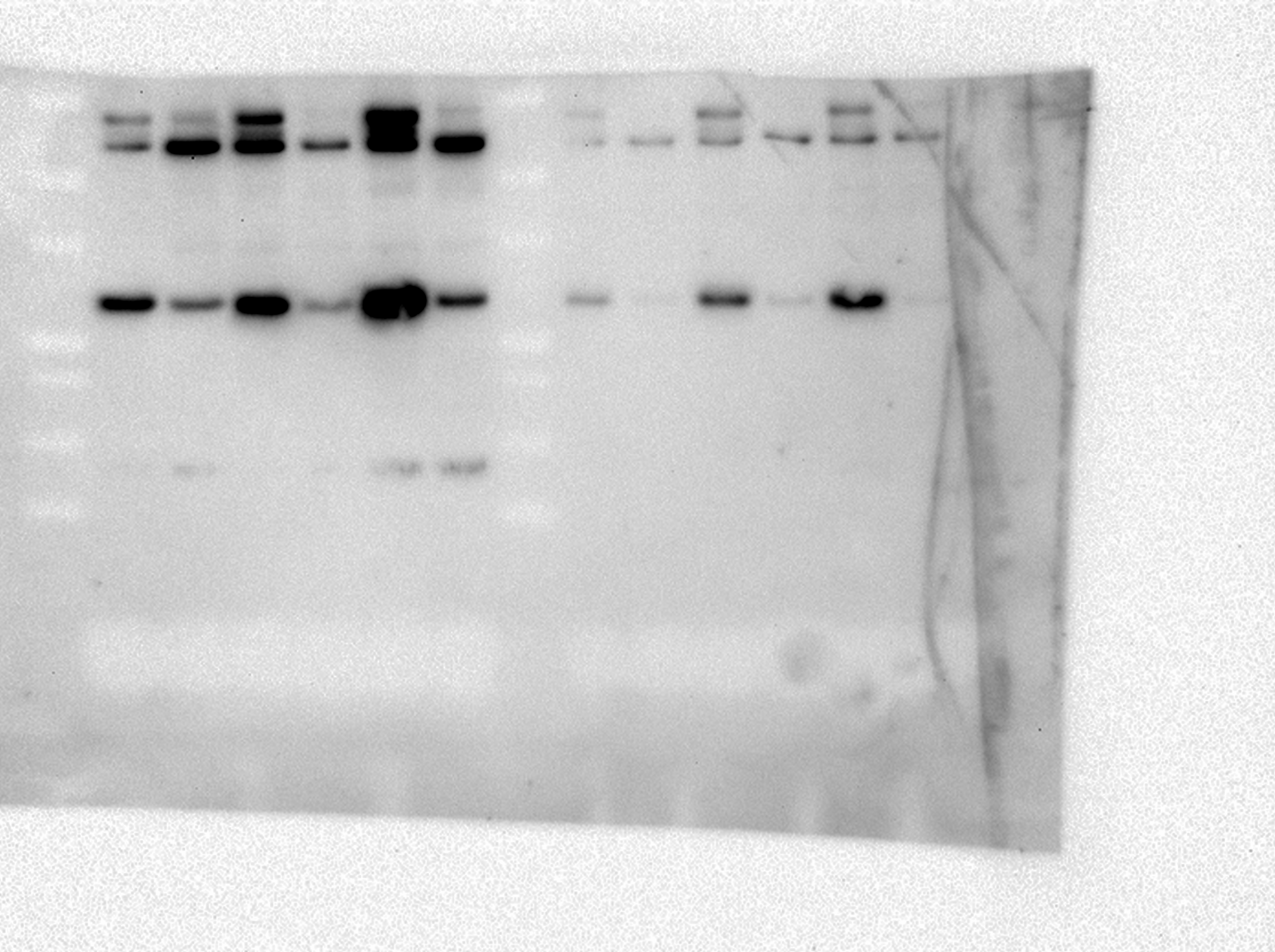

Supplement: Figure 3—source data 2. [file elife-102676-fig3-data2.zip › Donor 7-9/Fig3C-source data 2 Original files for western blot analysis displayed in Figure 3C.tif]

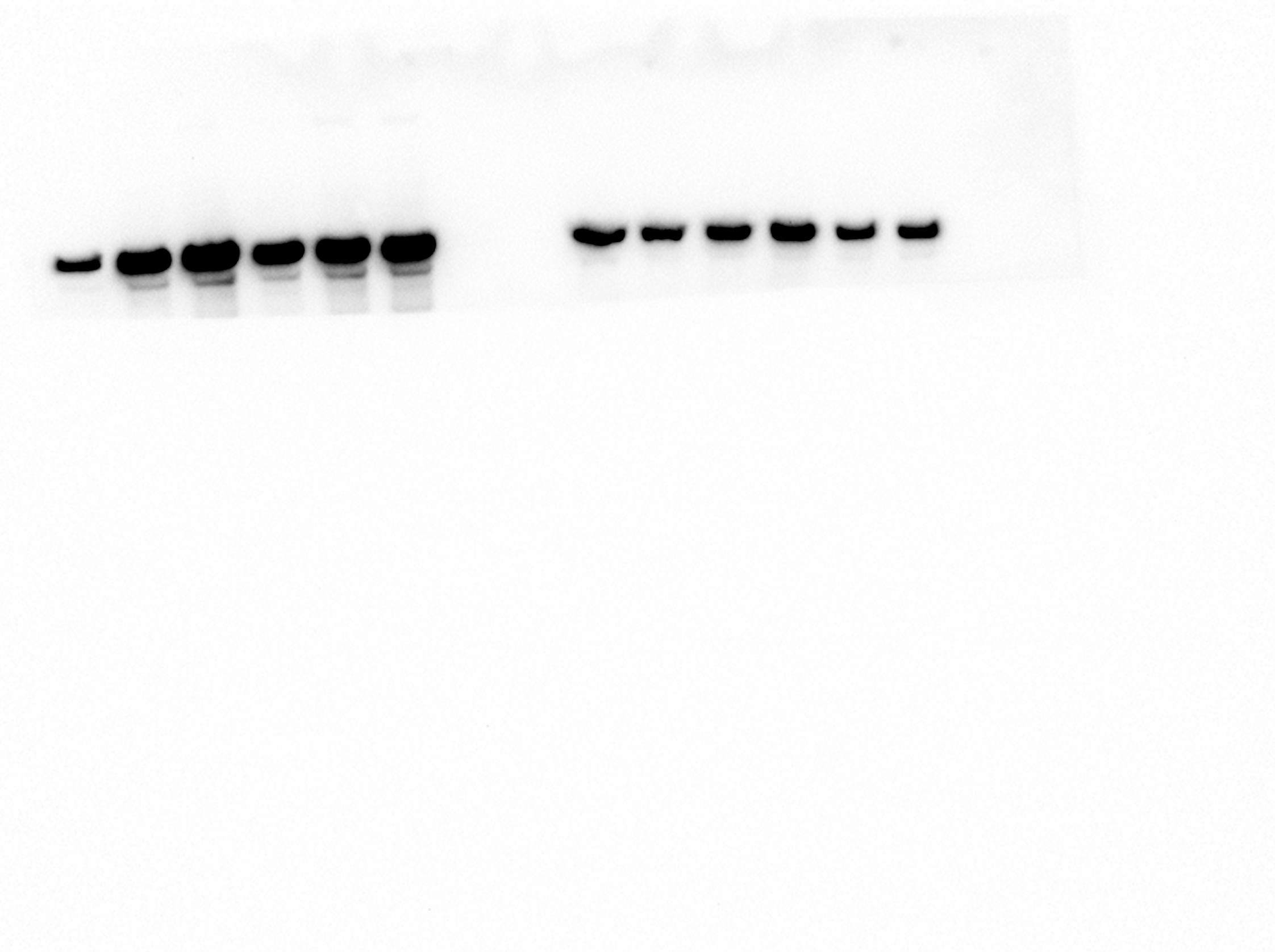

Supplement: Figure 3—source data 2. [file elife-102676-fig3-data2.zip › Donor 7-9/Fig3C-source data 3 Original files for western blot analysis displayed in Figure 3C.tif]

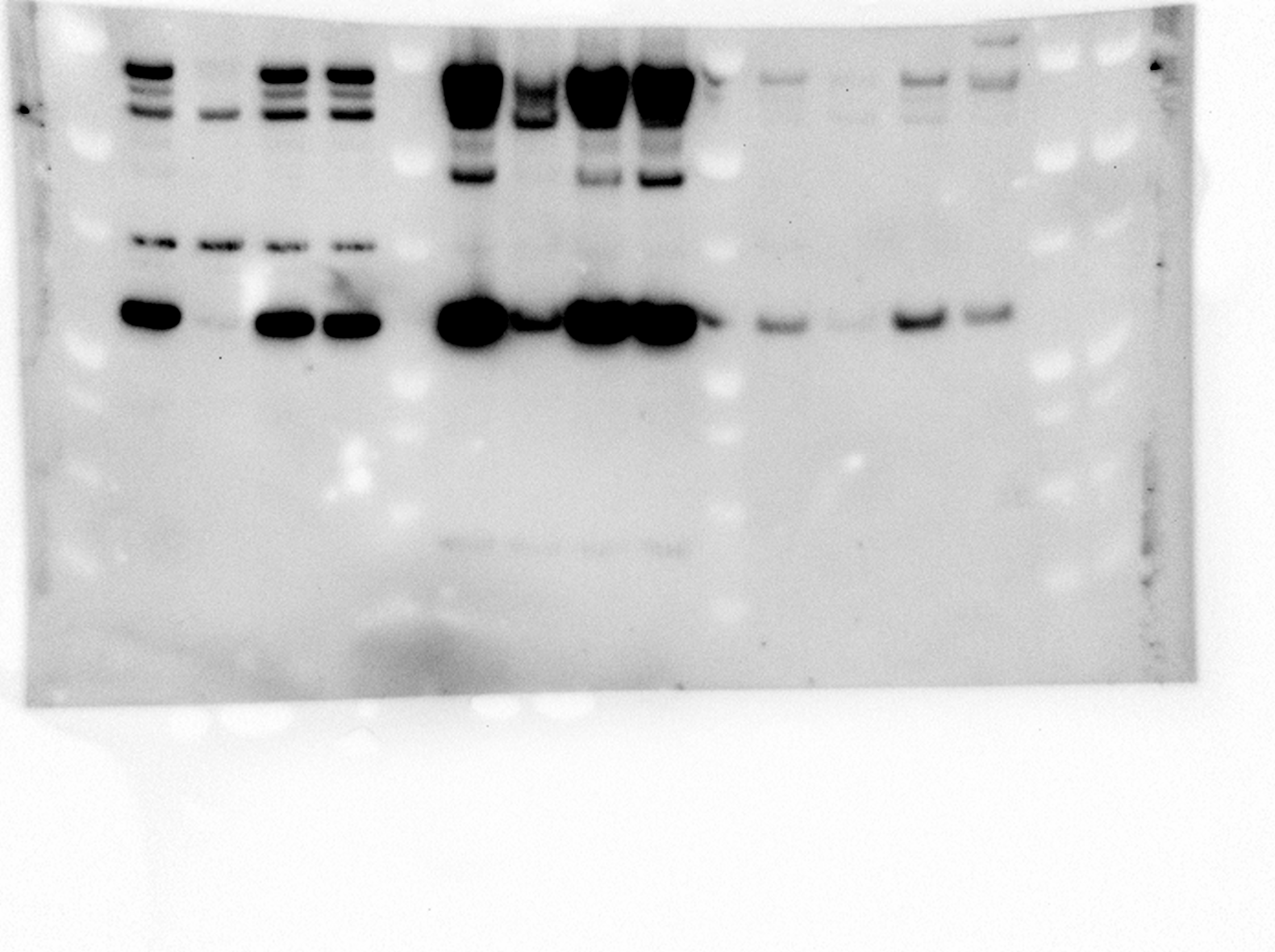

Supplement: Figure 3—source data 2. [file elife-102676-fig3-data2.zip › Donor 10-11/Fig3C-source data 4 Original files for western blot analysis displayed in Figure 3C.tif]

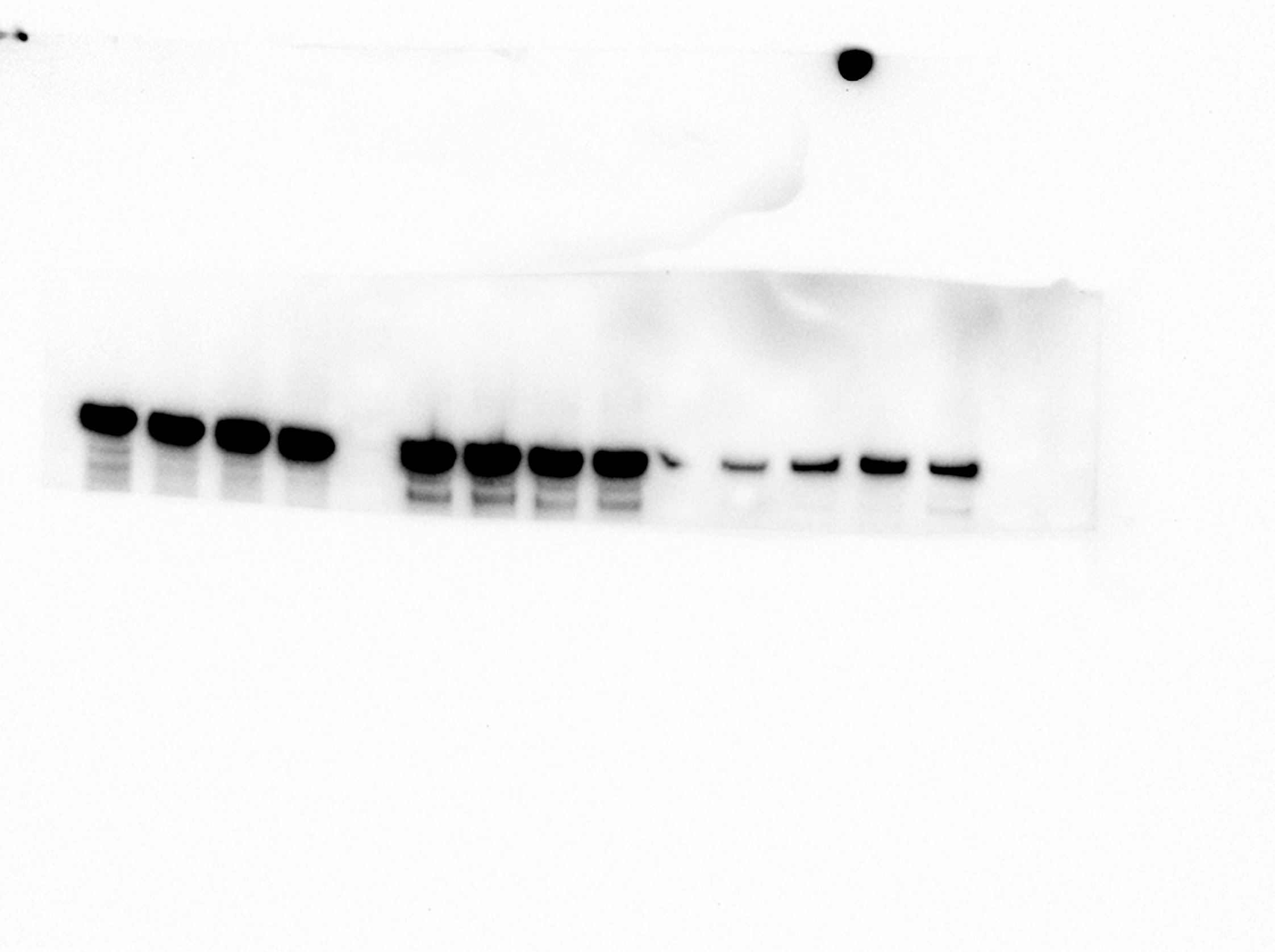

Supplement: Figure 3—source data 2. [file elife-102676-fig3-data2.zip › Donor 10-11/Fig3C-source data 5 Original files for western blot analysis displayed in Figure 3C.tif]

**Figure 4C: Primary CD4+ T cell:MDM coculture elicits CARD8-dependent inflammasome activation**

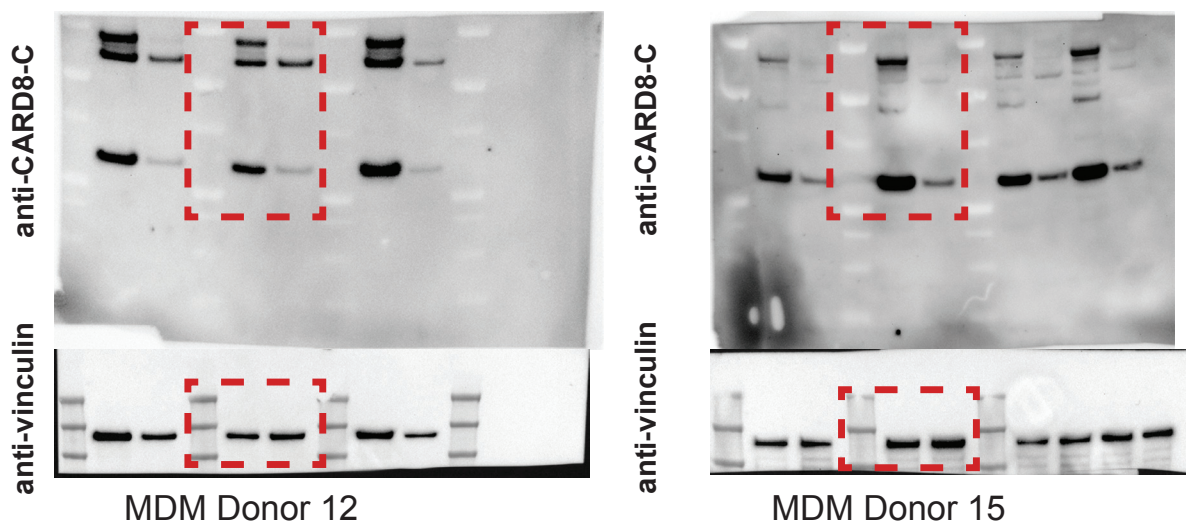

Supplement: Figure 4—source data 1. [file elife-102676-fig4-data1.pdf]

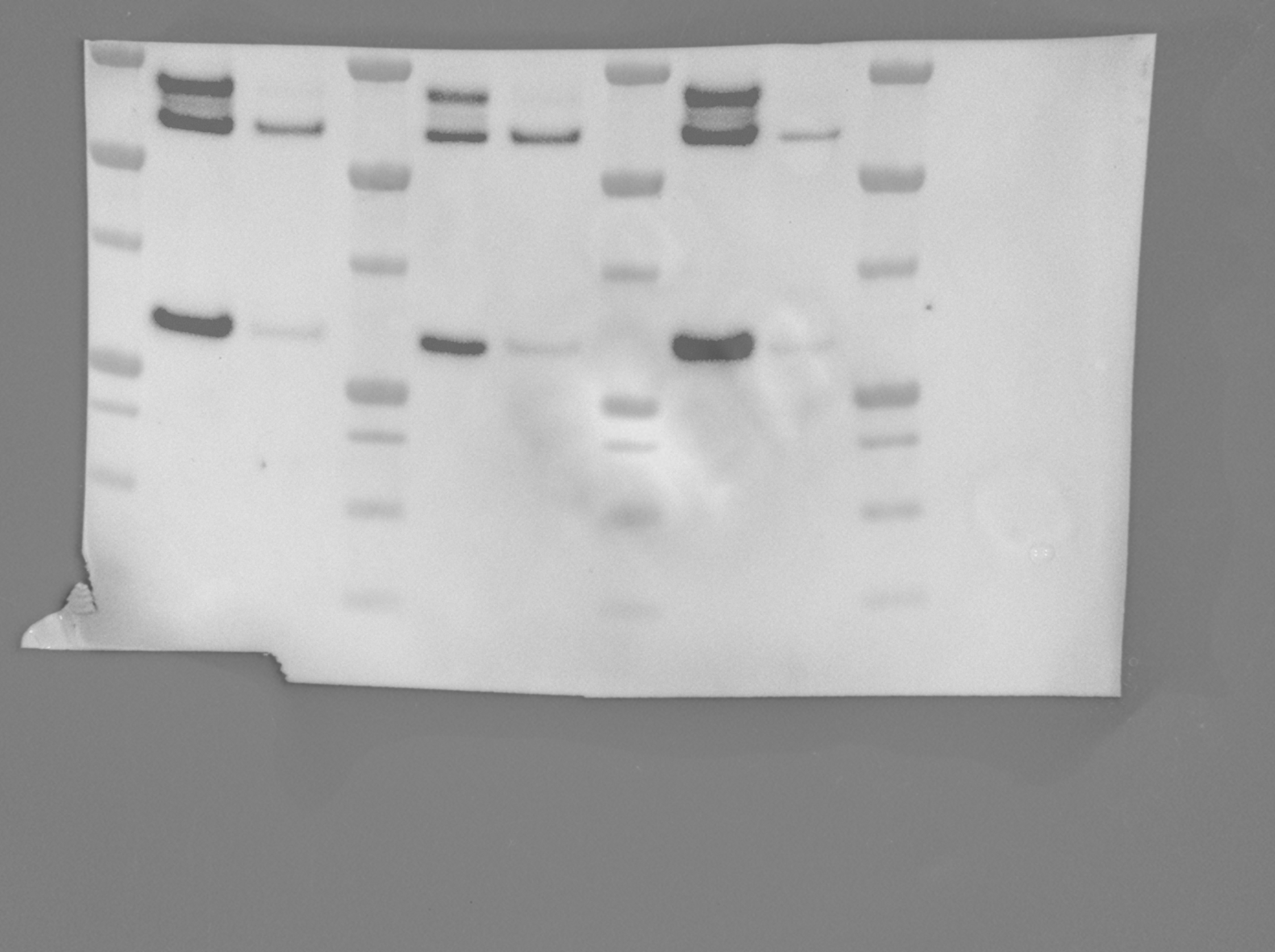

Supplement: Figure 4—source data 2. [file elife-102676-fig4-data2.zip › Fig4-source data 2 Original files for western blot analysis displayed in Figure 4C.tif]

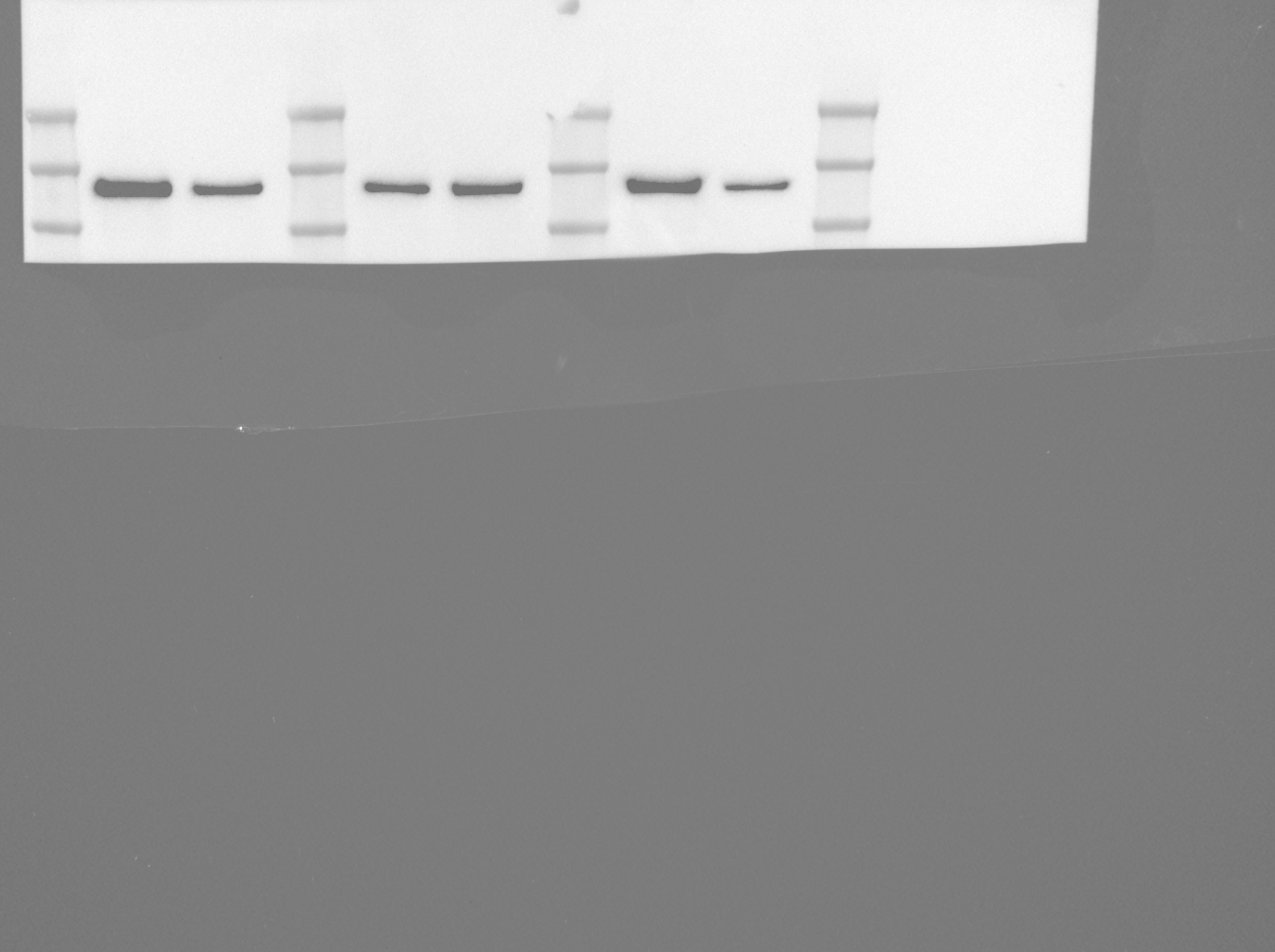

Supplement: Figure 4—source data 2. [file elife-102676-fig4-data2.zip › Fig4-source data 3 Original files for western blot analysis displayed in Figure 4C.tif]

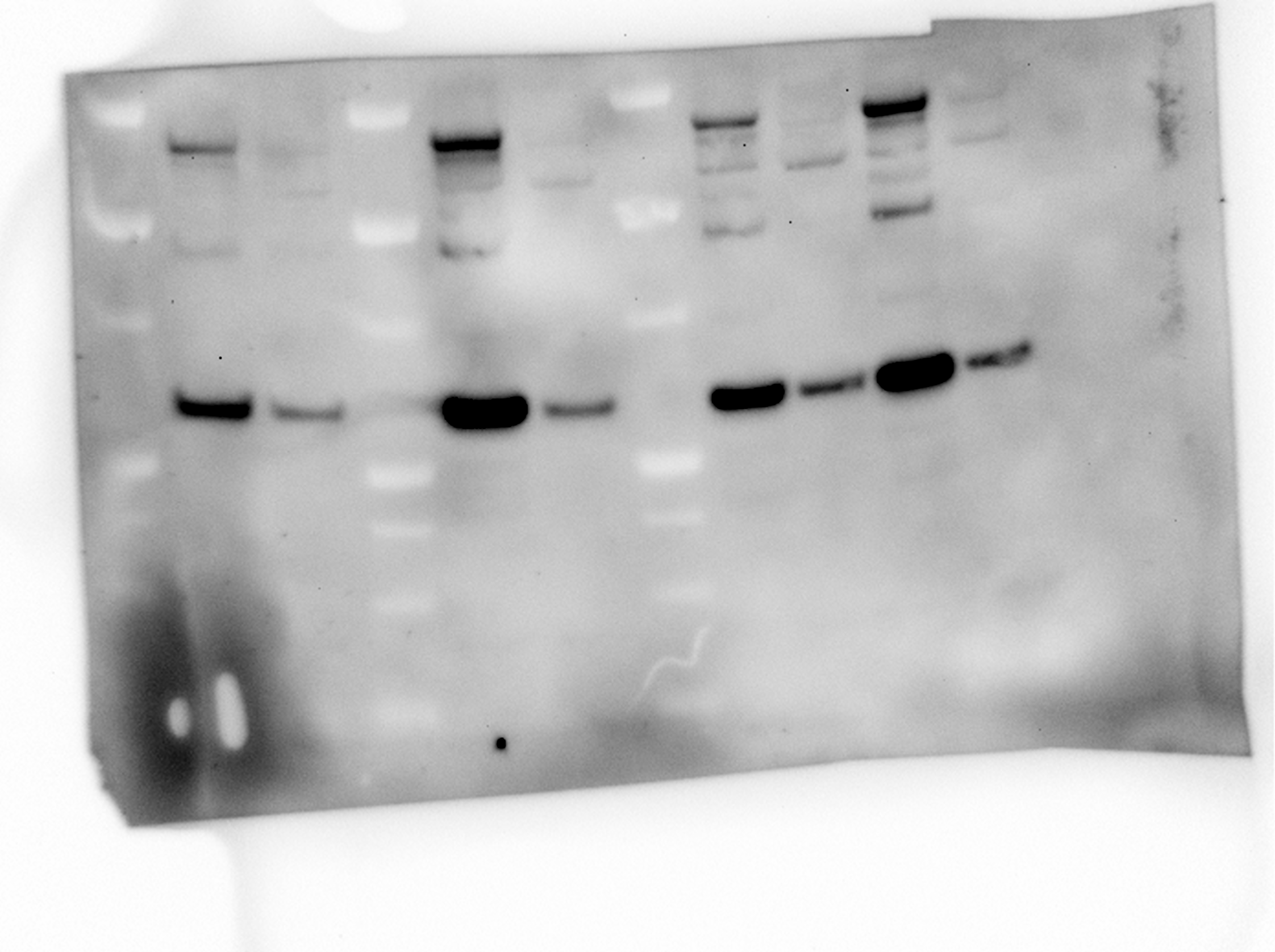

Supplement: Figure 4—source data 2. [file elife-102676-fig4-data2.zip › Fig4-source data 4 Original files for western blot analysis displayed in Figure 4C.tif]

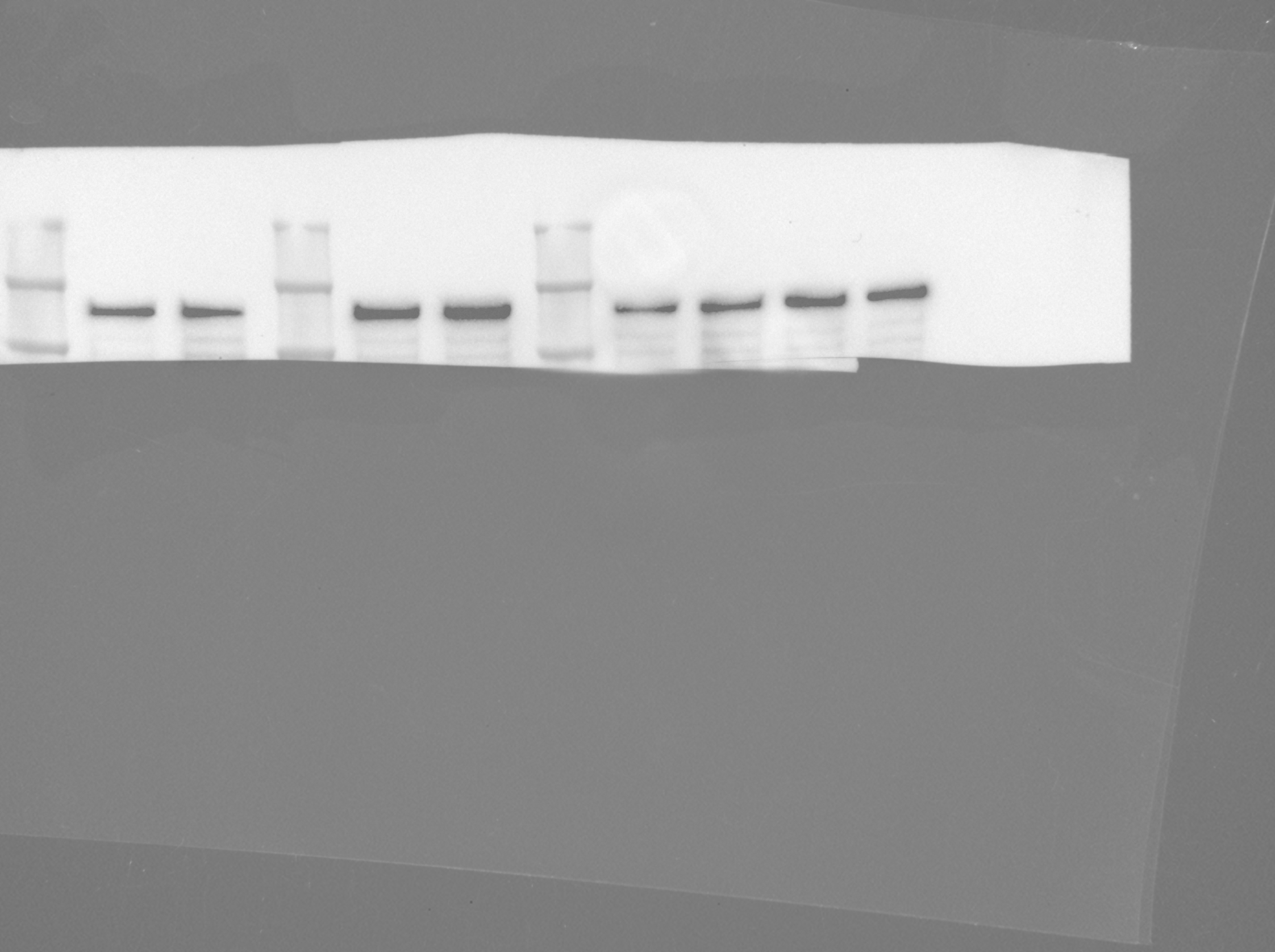

Supplement: Figure 4—source data 2. [file elife-102676-fig4-data2.zip › Fig4-source data 5 Original files for western blot analysis displayed in Figure 4C.tif]

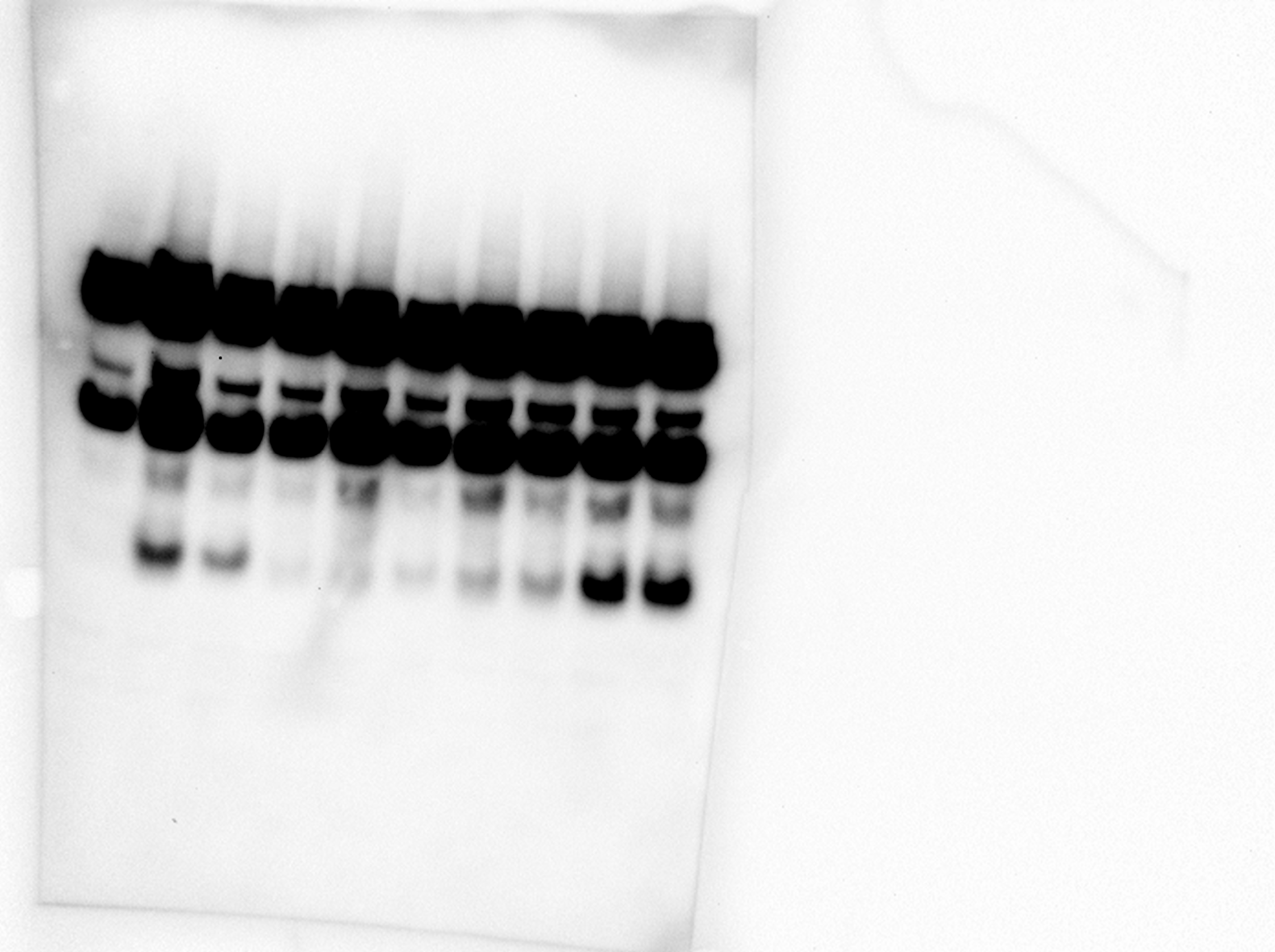

Supplement: Figure 5—source data 2. [file elife-102676-fig5-data2.zip › Fig5-source data 2 Original files for western blot analysis displayed in Figure 5A.tif]

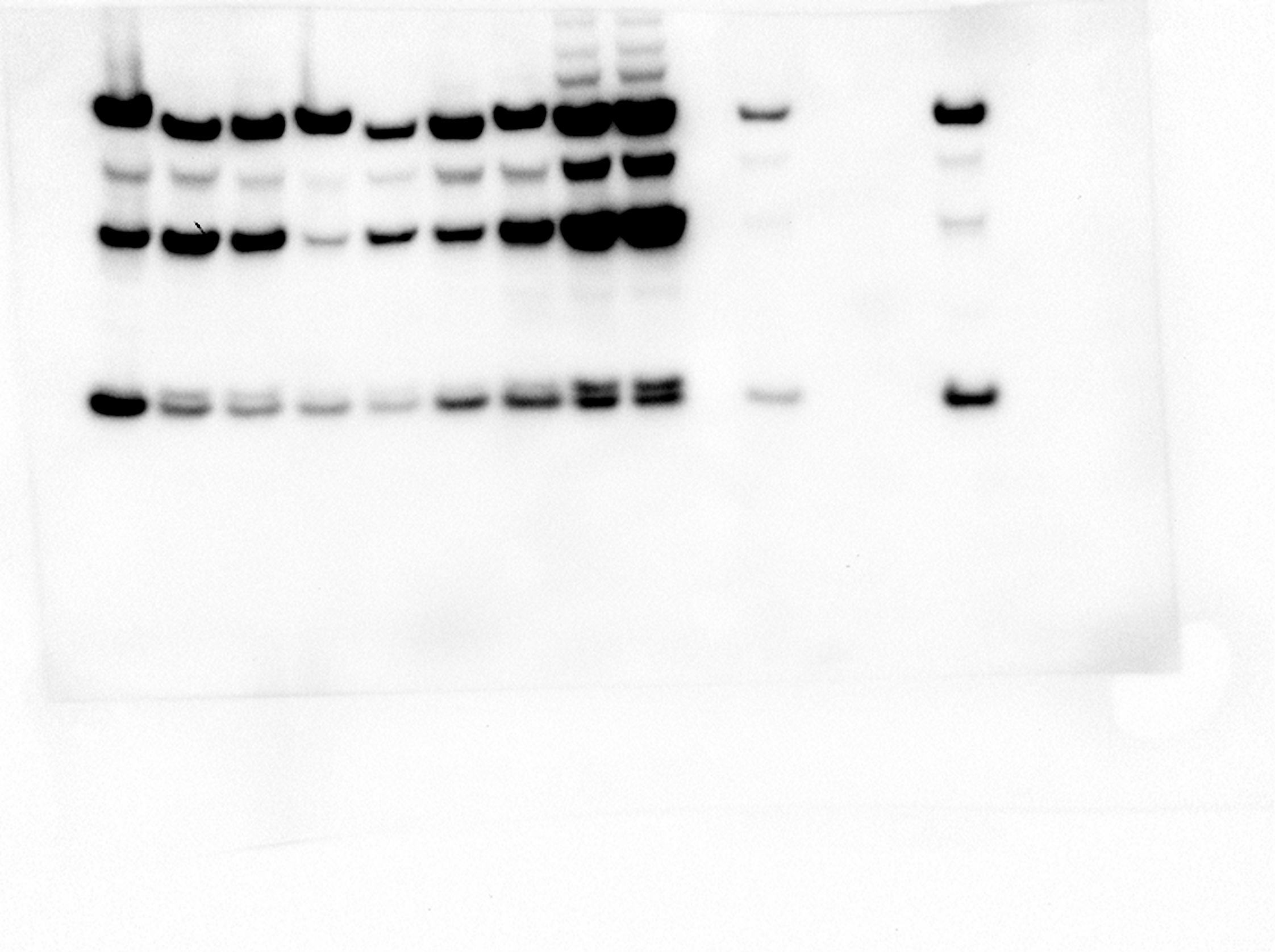

Supplement: Figure 5—source data 2. [file elife-102676-fig5-data2.zip › Fig5-source data 3 Original files for western blot analysis displayed in Figure 5A.tif]

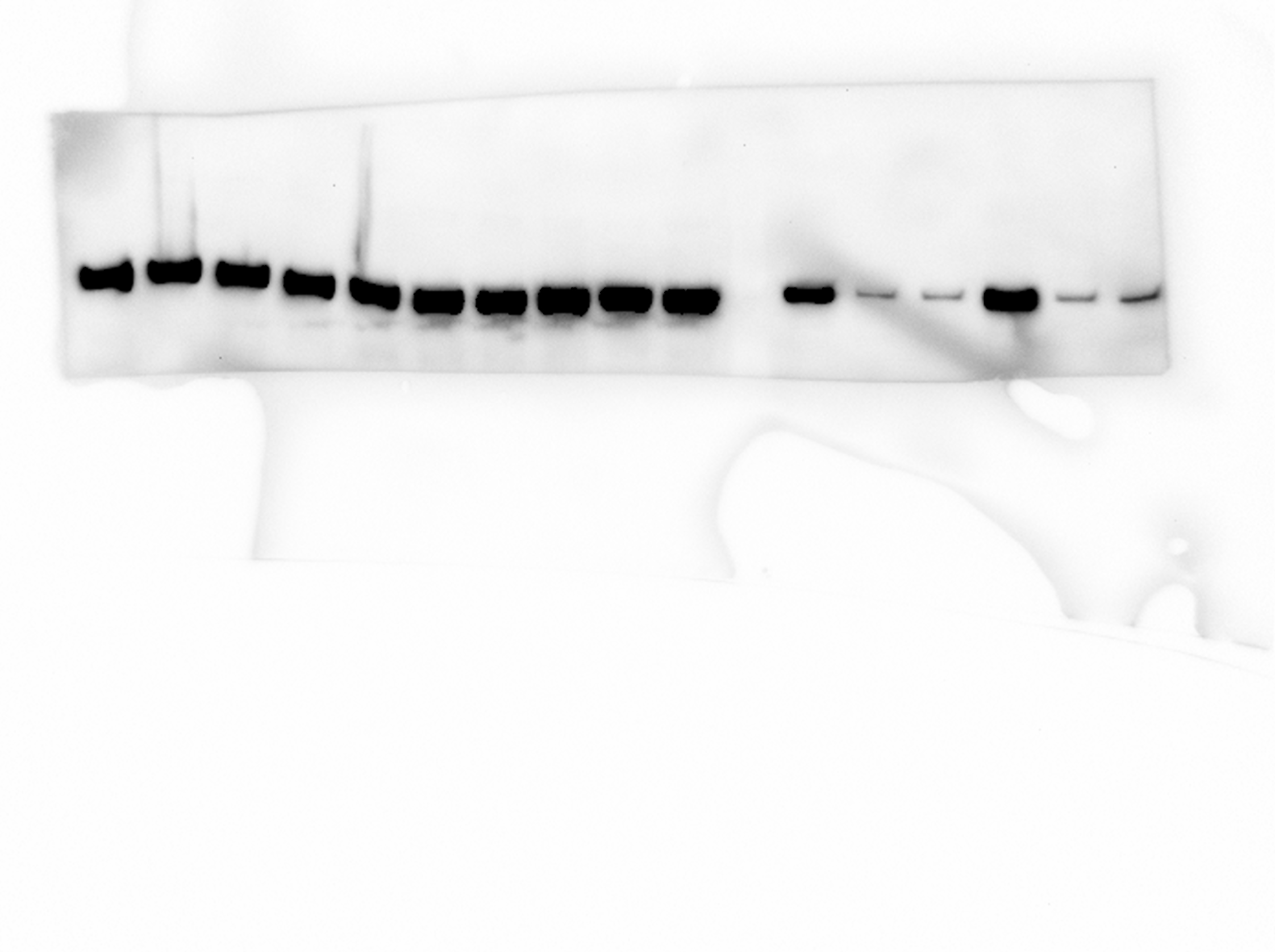

Supplement: Figure 5—source data 2. [file elife-102676-fig5-data2.zip › Fig5-source data 4 Original files for western blot analysis displayed in Figure 5A.tif]
