# Supplementary material for: CARD8 inflammasome activation during HIV-1 cell-to-cell transmission
Source: eLife. 2025 Jun 16;13:RP102676. doi: 10.7554/eLife.102676 (PMC12169848; doi:10.7554/eLife.102676)

Figure 5A: Protease inhibitor resistant strains of HIV-1 differentially cleave and activate CARD8

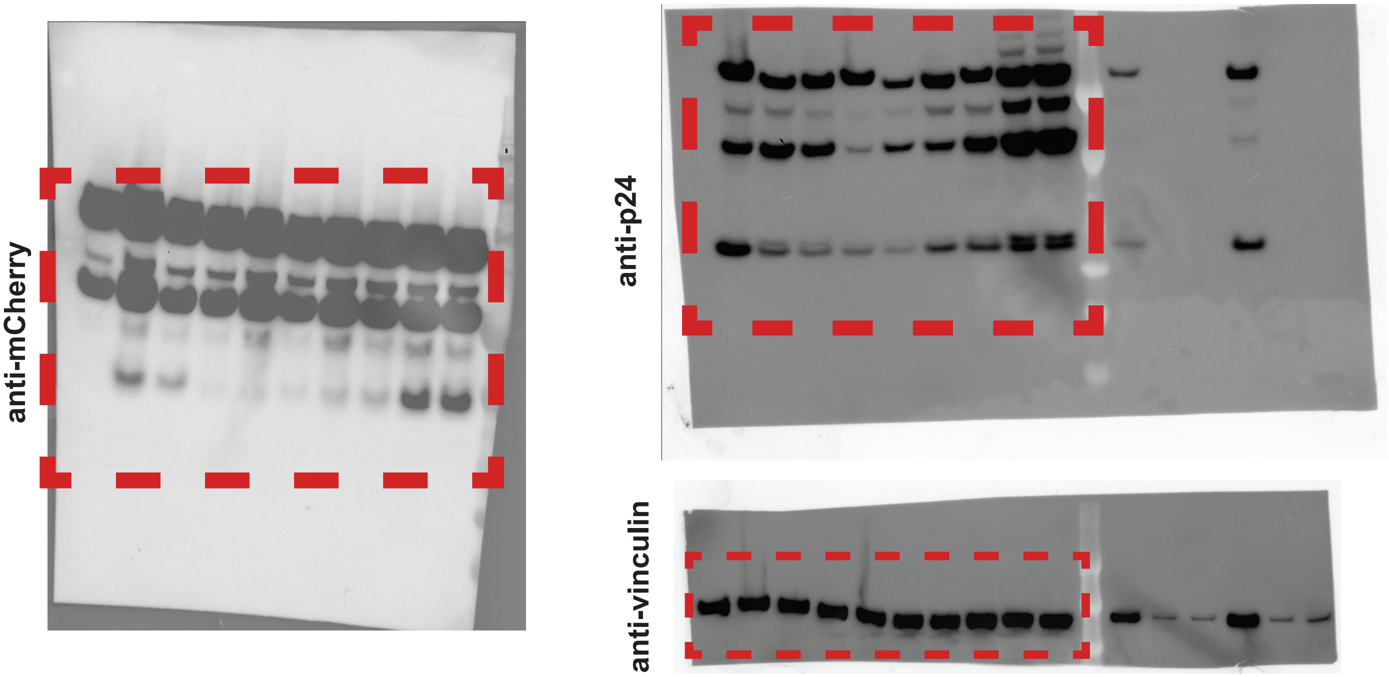

Supplement: Figure 5—source data 1. [file elife-102676-fig5-data1.pdf]
