# Supplementary material for: CARD8 inflammasome activation during HIV-1 cell-to-cell transmission
Source: eLife. 2025 Jun 16;13:RP102676. doi: 10.7554/eLife.102676 (PMC12169848; doi:10.7554/eLife.102676)
Supplement: Supplementary file 1. — 1These clones were previously cloned and assayed for PI-R in Varghese et al., 2013. The PI-R subset used in Figure 5B are bolded and highlighted in red or green and denote either hypo- or hyper-active CARD8 cleavage, respectively. The last column reports additional amino acid changes in the PI-R clones that were observed via whole plasmid Oxford Nanopore sequencing. *We were unable to sequence verify PI-R3 due to poor plasmid quality. NFV, nelfinavir; FPV, fosamprenavir; SQV. saquinavir; IDV, indinavir; LPV, lopinavir; TPV, tipranavir; DRV, darunavir. The consensus subtype B sequence can be found on the Stanford HIV Drug Resistance Database (HIVDB) (Stanford University HIV Drug Resistance Database, 2025). Relative CARD8 cleavage was determined by quantifying band volume of the CARD8 cleavage product in BioRad Image Lab 6 and comparing to cleavage with HIV-1LAI. [file elife-102676-supp1.docx]

**Table S1: Protease inhibitor resistance mutations and relative CARD8 cleavage.**

| **Clone name** | **Reported PI-resistance  mutations in HIV^PR^** | **HIV^gag^ mutation** | **Strongest**  **PI-R^1^** | **% CARD8 cleavage**  **(relative to HIV_LAI_)** | **Additional amino acid changes in HIV^PR^ relative to NL4.3** |
| --- | --- | --- | --- | --- | --- |
| HIV-1_LAI_ | wildtype | wildtype | wildtype | 100 | 37S |
| CA126802  (PI-R1) | 11I, 32I, 33F, 46I, 47V, 54M, 58E, 73S, 84V, 89V, 90M | 431V (NC/p1)  437N (NC/p1)  453LF (p1/p6) | FPV, LPV, TPV, DRV | 51 | 10I, 12K, 13V, 20V, 35G, 36I, 37D, 57K, 63P, 64V, 66V, 7IV |
| **CA122805 (PI-R2)** | **10F, 33F, 43T, 46L, 54V, 82A, 84V, 90M** | **431V (NC/p1)**  **532S (p1/p6)** | **SQV** | **7** | 16A,19I, 20R, 35D, 36L, 37D, 55R, 57K, 60E, 62V, 63P, 71V, 93L |
| CA126805 (PI-R3) | 33F, 43T, 46I, 48V, 50V, 54S, 82A | 437N (NC/p1)  449VF (p1/p6)  PTAP insertion (p6) | SQV, IDV, LPV | **8** | not determined* |
| CA96457  (PI-R5) | 48V, 53L, 54V, 82A, 90M | 436R  (NC/p1) | SQV | 11 | 10I, 37D, 63P, 71T, 77I, 93L |
| **CA96458**  **(PI-R9)** | **10F, 30N, 33F, 43T, 84V, 88D, 90M** | **431V (NC/p1)** | **NFV, SQV** | **24** | 15V, 35D, 36L, 37E, 60E, 62V, 63P |
| CA50834-1  (PI-R10) | 24I, 46L, 54V, 76V, 82A | 431V (NC/p1) | IDV, LPV | 38 | 10I, 14R, 35D, 36I, 37E, 63P,71V |
| **CA96451**  **(PI-R12)** | **32I, 33F, 43T, 46I, 47V, 54M, 73S, 82A, 89V, 90M** | **437N (NC/p1)**  **PTAP insertion (p6)** | **FPV, LPV** | **192** | 10V, 12V, 13V, 15V, 20M, 60E, 61N, 62V, 63P, 67Y, 69K, 71I, 72L, 77I |
| **CA20392-1**  **(PI-R13)** | **24I, 46L, 54V, 82A** | **431V (NC/p1)** | **LPV** | **206** | 10I, 14R, 35D, 36I, 37E, 63P, 71V |
