# Supplementary material for: CARD8 inflammasome activation during HIV-1 cell-to-cell transmission
Source: eLife. 2025 Jun 16;13:RP102676. doi: 10.7554/eLife.102676 (PMC12169848; doi:10.7554/eLife.102676)
Supplement: Supplementary file 2. [file elife-102676-supp2.docx]

**Supplementary File 2:**

| **sgRNA** | **Sequence** |
| --- | --- |
| CARD8 sgRNA1 | CUCUGCAGUGACAUCAAACA |
| CARD8 sgRNA2 | UGACGAUUGCGUUUGGUUCC |
| CARD8 sgRNA3 | AGCGUUUGGUUCCCCACUGC |
| AAVS1 sgRNA 1 | GUUAAUGUGGCUCUGGUUCU |
| AAVS1 sgRNA 2 | ACCCCACAGUGGGGCCACUA |
| AAVS1 sgRNA 3 | CCUUCCUAGUCUCCUGAUAU |
| NLRP3 sgRNA 1 | GCUCAGAAUGCUCAUCAUCG |
| NLRP3 sgRNA 2 | GAUGAUGUUGGACUGGGCAU |
| NLRP3 sgRNA 3 | CAAGGCUCACCUCCCGACAG |
